# Diethyl 2,5-bis­[(2,3-dihydro­thieno[3,4-*b*][1,4]dioxin-5-yl)methyl­idene­amino]­thio­phene-3,4-dicarboxyl­ate acetone monosolvate

**DOI:** 10.1107/S160053681104339X

**Published:** 2011-11-02

**Authors:** Stéphane Dufresne, Andréanne Bolduc, W. G. Skene

**Affiliations:** a Department of Chemistry, University of Montreal, CP 6128, succ. Centre-ville, Montréal, Québec, Canada H3C 3J7

## Abstract

The unique 3,4-ethyl­ene­dioxy­thio­phene (EDOT) unit of the title compound, C_24_H_22_N_2_O_8_S_3_·C_3_H_6_O, is twisted by 1.9 (3)° relative to the central thio­phene ring. The three heterocyclic units are anti­periplanar. In the crystal, inversion dimers linked by pairs of C—H⋯O hydrogen bonds connect the heterocycles. π–π interactions occur between the central thiophene and the imine bond of the molecule [distance between the ring centroid of the ring and the azomethine bond = 3.413 (3) Å.

## Related literature

For general background, see: Dufresne *et al.* (2007 ▶). For related structures, see: Dufresne *et al.* (2006 ▶). For π–π inter­actions, see: Janiak (2000 ▶).
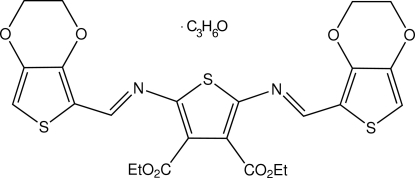


## Experimental

### 

#### Crystal data

C_24_H_22_N_2_O_8_S_3_·C_3_H_6_O
*M* *_r_* = 620.69
Monoclinic, 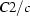

*a* = 13.288 (3) Å
*b* = 23.541 (5) Å
*c* = 9.0627 (18) Å
β = 98.27 (3)°
*V* = 2805.5 (10) Å^3^
*Z* = 4
Cu *K*α radiation
μ = 2.91 mm^−1^
*T* = 150 K
0.25 × 0.10 × 0.04 mm

#### Data collection

Bruker SMART 6000 diffractometer
Absorption correction: multi-scan (*SADABS*; Sheldrick, 1996 ▶) *T*_min_ = 0.530, *T*_max_ = 0.892
16861 measured reflections
2718 independent reflections
2215 reflections with *I* > 2σ(*I*)
*R*_int_ = 0.060

#### Refinement

*R*[*F* ^2^ > 2σ(*F* ^2^)] = 0.038
*wR*(*F* ^2^) = 0.101
*S* = 1.05
2718 reflections
189 parameters
H-atom parameters constrained
Δρ_max_ = 0.25 e Å^−3^
Δρ_min_ = −0.37 e Å^−3^

### 

Data collection: *SMART* (Bruker, 2003 ▶); cell refinement: *SMART*; data reduction: *SAINT* (Bruker, 2004 ▶); program(s) used to solve structure: *SHELXS97* (Sheldrick, 2008 ▶); program(s) used to refine structure: *SHELXL97* (Sheldrick, 2008 ▶); molecular graphics: *SHELXTL* (Sheldrick, 2008 ▶); software used to prepare material for publication: *UdMX* (Marris, 2004 ▶).

## Supplementary Material

Crystal structure: contains datablock(s) I, global. DOI: 10.1107/S160053681104339X/mw2024sup1.cif

Structure factors: contains datablock(s) I. DOI: 10.1107/S160053681104339X/mw2024Isup2.hkl

Supplementary material file. DOI: 10.1107/S160053681104339X/mw2024Isup3.cml

Additional supplementary materials:  crystallographic information; 3D view; checkCIF report

## Figures and Tables

**Table 1:** Hydrogen-bond geometry (Å, °)

*D*—H⋯*A*	*D*—H	H⋯*A*	*D*⋯*A*	*D*—H⋯*A*
C1—H1⋯O1^i^	0.95	2.58	3.514 (3)	168 (2)

Symmetry code: (i) 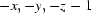.

**Fig. 1.**
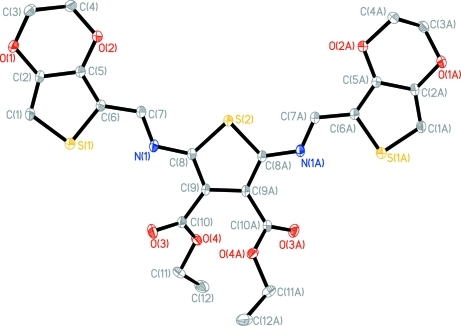
ORTEP representation of (I) with the numbering scheme adopted (SHELXTL; Sheldrick, 2008). Ellipsoids drawn at 30% probability level. Solvent molecules and hydrogens were omitted for clarity.

**Fig. 2.**
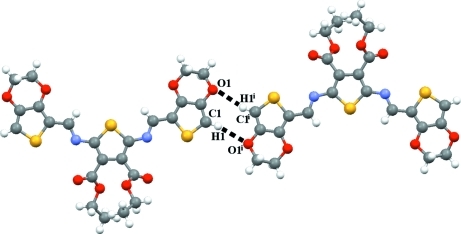
Supramolecular structure showing the intermolecular H-bonding giving the structural arrangement. Dashed lines indicate hydrogen bonds. Hydrogens were omitted for clarity. [Symmetry codes: (i) -x, -y, -1-z].

## supplementary crystallographic information

### Comment

Compound (I) was prepared during our on-going research on conjugated azomethines. The title compound is one of a limited number of reported crystal structures of 3,4-ethylenedioxythiophene (EDOT) azomethine derivatives. The structure was confirmed by the X-ray crystal structure and, as shown in Fig. 1, the azomethine bond adopts the thermodynamically stable E isomer. Both the title compound and the molecule of acetone solvent crystallized within the lattice have crystallographically imposed *C_2_* symmetry.

A major point of interest is the azomethine bond. The bond lengths for C6—C7, N1—C7 and N1—C8 are 1.430 (2), 1.287 (2) and 1.378 (2) Å, respectively. The bond distances are consistent with those of similar compounds consisting uniquely of thiophenes with two azomethine bonds (Dufresne *et al.*, 2006). The analogous bond lengths for the all-thiophene counterpart are: 1.441 (4), 1.272 (3) and 1.388 (3) Å.

It was found that the azomethine bond is nearly coplanar the terminal EDOT and the central thiophene. Thus the angle between the planes defined by S1, C1, C2, C5, C6 and S2, C8, C8B, C9, C9B is 1.9 (3)°. This is smaller than that in the all-thiophene analogue where the terminal thiophenes are twisted by 9.04 (4)° and 25.07 (6)° from the central thiophene.

The three-dimensional network of (I) involves multiple interactions that are in part responsible for the molecular organization of the crystal lattice. As can be seen in Fig. 2, there is a pair-wise supramolecular arrangement involving a C—H···O interaction between C1H1 in one EDOT and O1 in the EDOT at -x, -y, -1-z (Table 1). This donor–acceptor arrangement leads to a linear and planar organization between different molecules within the lattice. There are also π–π interactions between the thiophene ring containing S1, C1, C2, C5, C6 and the centroid of the azomethine bond (C7, N1) in the molecule at 1-x, -y, -z. The distance between the centroids of the ring and the azomethine bond was found to be 3.413 (3) Å. This separation is in the middle of the range associated with π–π stacking interactions (Janiak, 2000). The organization of the molecules of (I) within in the lattice is aligned in a ladder-type orientation.

### Experimental

2,3-dihydrothieno[3,4-*b*][1,4]dioxine-5-carbaldehyde (112.3 mg, 0.66 mmol) and diethyl 2,5-diaminothiophene-3,4-dicarboxylate (77.3 mg, 0.3 mmol) were dissolved in toluene (20 ml) followed by the addition of DABCO (268 mg, 2.4 mmol) and titanium tetrachloride (1 *M*, 0.6 ml). The reaction mixture was refluxed for 3 h, concentrated, and then re-dissolved in acetone. The solution was filtered and the solvent was evaporated. The crude product was loaded onto a silica column and eluted with hexanes/ethyl acetate, up to (70%/30% *v*/*v*). The product was a red solid (169 mg, 70%). Single crystals of (I) were obtained by slow evaporation of an acetone solution of (I).

### Refinement

H atoms were placed in calculated positions (C—H = 0.93–0.97 Å) and included in the refinement in the riding-model approximation, with *U*_iso_(H) = 1.2*U*_eq_(C).

### Crystal data

**Table:**

C_24_H_22_N_2_O_8_S_3_·C_3_H_6_O	*F*(000) = 1296
*M**_r_* = 620.69	*D*_x_ = 1.470 Mg m^−^^3^
Monoclinic, *C*2/*c*	Cu *K*α radiation, λ = 1.54178 Å
Hall symbol: -C 2yc	Cell parameters from 25 reflections
*a* = 13.288 (3) Å	θ = 15.0–30.0°
*b* = 23.541 (5) Å	µ = 2.91 mm^−^^1^
*c* = 9.0627 (18) Å	*T* = 150 K
β = 98.27 (3)°	Needle, red
*V* = 2805.5 (10) Å^3^	0.25 × 0.10 × 0.04 mm
*Z* = 4	

### Data collection

**Table:**

Bruker SMART 6000 diffractometer	2718 independent reflections
Radiation source: Rotating anode	2215 reflections with *I* > 2σ(*I*)
Montel 200 optics	*R*_int_ = 0.060
Detector resolution: 5.5 pixels mm^-1^	θ_max_ = 72.0°, θ_min_ = 3.8°
ω scans	*h* = −16→16
Absorption correction: multi-scan (*SADABS*; Sheldrick, 1996)	*k* = −28→28
*T*_min_ = 0.530, *T*_max_ = 0.892	*l* = −8→10
16861 measured reflections	

### Refinement

**Table:**

Refinement on *F*^2^	Primary atom site location: structure-invariant direct methods
Least-squares matrix: full	Secondary atom site location: difference Fourier map
*R*[*F*^2^ > 2σ(*F*^2^)] = 0.038	Hydrogen site location: inferred from neighbouring sites
*wR*(*F*^2^) = 0.101	H-atom parameters constrained
*S* = 1.05	*w* = 1/[σ^2^(*F*_o_^2^) + (0.0584*P*)^2^ + 0.6834*P*] where *P* = (*F*_o_^2^ + 2*F*_c_^2^)/3
2718 reflections	(Δ/σ)_max_ < 0.001
189 parameters	Δρ_max_ = 0.25 e Å^−^^3^
0 restraints	Δρ_min_ = −0.37 e Å^−^^3^

### Special details

**Table:**

Geometry. All e.s.d.'s (except the e.s.d. in the dihedral angle between two l.s. planes) are estimated using the full covariance matrix. The cell e.s.d.'s are taken into account individually in the estimation of e.s.d.'s in distances, angles and torsion angles; correlations between e.s.d.'s in cell parameters are only used when they are defined by crystal symmetry. An approximate (isotropic) treatment of cell e.s.d.'s is used for estimating e.s.d.'s involving l.s. planes.
Refinement. Refinement of *F*^2^ against ALL reflections. The weighted *R*-factor *wR* and goodness of fit *S* are based on *F*^2^, conventional *R*-factors *R* are based on *F*, with *F* set to zero for negative *F*^2^. The threshold expression of *F*^2^ > σ(*F*^2^) is used only for calculating *R*-factors(gt) *etc*. and is not relevant to the choice of reflections for refinement. *R*-factors based on *F*^2^ are statistically about twice as large as those based on *F*, and *R*-factors based on ALL data will be even larger.

### Fractional atomic coordinates and isotropic or equivalent isotropic displacement parameters (Å^2^)

**Table:**

	*x*	*y*	*z*	*U*_iso_*/*U*_eq_	
S2	0.5000	0.01366 (3)	0.2500	0.02556 (17)	
S1	0.18156 (4)	0.05553 (2)	−0.22135 (6)	0.02949 (15)	
O1	0.06743 (10)	−0.08612 (6)	−0.39079 (15)	0.0293 (3)	
O4	0.39123 (10)	0.20642 (5)	0.24719 (14)	0.0283 (3)	
O2	0.23045 (10)	−0.10493 (6)	−0.14440 (15)	0.0302 (3)	
C8	0.42699 (13)	0.06598 (8)	0.1453 (2)	0.0231 (4)	
N1	0.34663 (11)	0.05550 (7)	0.03454 (18)	0.0257 (4)	
C6	0.24046 (13)	−0.00282 (8)	−0.1303 (2)	0.0245 (4)	
O3	0.37191 (11)	0.18269 (6)	0.00449 (15)	0.0357 (4)	
C5	0.19872 (14)	−0.05222 (8)	−0.1925 (2)	0.0242 (4)	
C4	0.15966 (15)	−0.14861 (8)	−0.2055 (2)	0.0314 (5)	
H4A	0.1004	−0.1489	−0.1506	0.038*	
H4B	0.1931	−0.1862	−0.1923	0.038*	
C9	0.45815 (13)	0.11955 (8)	0.1910 (2)	0.0226 (4)	
C10	0.40336 (13)	0.17185 (8)	0.1330 (2)	0.0250 (4)	
C7	0.32136 (13)	0.00486 (8)	−0.0096 (2)	0.0252 (4)	
H7	0.3560	−0.0271	0.0374	0.030*	
C11	0.34758 (16)	0.26181 (8)	0.2084 (2)	0.0349 (5)	
H11A	0.2762	0.2580	0.1601	0.042*	
H11B	0.3868	0.2817	0.1391	0.042*	
C1	0.10182 (15)	0.01334 (8)	−0.3404 (2)	0.0292 (4)	
H1	0.0514	0.0275	−0.4165	0.035*	
C2	0.11942 (14)	−0.04248 (8)	−0.3127 (2)	0.0249 (4)	
C3	0.12391 (15)	−0.13842 (8)	−0.3688 (2)	0.0311 (5)	
H3A	0.1834	−0.1367	−0.4231	0.037*	
H3B	0.0803	−0.1704	−0.4100	0.037*	
C12	0.3528 (2)	0.29398 (10)	0.3526 (3)	0.0482 (6)	
H12A	0.3162	0.2729	0.4215	0.072*	
H12B	0.3215	0.3315	0.3334	0.072*	
H12C	0.4240	0.2986	0.3970	0.072*	
O91	0.0000	0.74454 (10)	0.7500	0.0691 (8)	
C92	0.0000	0.69316 (13)	0.7500	0.0366 (7)	
C91	−0.07780 (18)	0.65932 (12)	0.6544 (3)	0.0545 (7)	
H91A	−0.1199	0.6388	0.7172	0.082*	
H91B	−0.0441	0.6320	0.5961	0.082*	
H91C	−0.1209	0.6847	0.5867	0.082*	

### Atomic displacement parameters (Å^2^)

**Table:**

	*U*^11^	*U*^22^	*U*^33^	*U*^12^	*U*^13^	*U*^23^
S2	0.0214 (3)	0.0267 (4)	0.0269 (4)	0.000	−0.0023 (2)	0.000
S1	0.0292 (3)	0.0272 (3)	0.0296 (3)	−0.00025 (19)	−0.0042 (2)	−0.00046 (18)
O1	0.0256 (7)	0.0320 (8)	0.0279 (8)	−0.0038 (6)	−0.0040 (6)	−0.0044 (6)
O4	0.0320 (7)	0.0265 (7)	0.0253 (8)	0.0075 (6)	0.0003 (5)	0.0018 (5)
O2	0.0288 (7)	0.0267 (7)	0.0321 (8)	−0.0010 (6)	−0.0062 (6)	0.0006 (6)
C8	0.0180 (9)	0.0294 (10)	0.0213 (10)	0.0014 (7)	0.0009 (7)	−0.0003 (7)
N1	0.0190 (8)	0.0323 (9)	0.0241 (9)	0.0003 (6)	−0.0024 (6)	−0.0027 (6)
C6	0.0201 (9)	0.0284 (10)	0.0241 (10)	−0.0005 (7)	0.0003 (7)	−0.0010 (7)
O3	0.0384 (8)	0.0421 (9)	0.0236 (8)	0.0084 (6)	−0.0057 (6)	0.0033 (6)
C5	0.0210 (9)	0.0283 (10)	0.0229 (10)	0.0000 (7)	0.0017 (7)	0.0011 (7)
C4	0.0288 (10)	0.0268 (10)	0.0374 (12)	−0.0023 (8)	0.0012 (8)	−0.0024 (8)
C9	0.0173 (9)	0.0292 (10)	0.0202 (10)	−0.0008 (7)	−0.0009 (7)	−0.0011 (7)
C10	0.0190 (9)	0.0304 (10)	0.0245 (10)	−0.0014 (7)	−0.0008 (7)	0.0003 (8)
C7	0.0209 (9)	0.0306 (10)	0.0235 (11)	0.0002 (8)	0.0009 (7)	0.0000 (8)
C11	0.0380 (11)	0.0282 (11)	0.0386 (13)	0.0087 (9)	0.0054 (9)	0.0086 (9)
C1	0.0263 (10)	0.0340 (11)	0.0250 (11)	−0.0011 (8)	−0.0040 (8)	−0.0003 (8)
C2	0.0193 (9)	0.0332 (10)	0.0212 (10)	−0.0027 (7)	−0.0002 (7)	−0.0034 (8)
C3	0.0296 (10)	0.0292 (11)	0.0337 (12)	−0.0035 (8)	0.0019 (8)	−0.0068 (8)
C12	0.0624 (16)	0.0313 (12)	0.0499 (16)	0.0116 (11)	0.0047 (12)	−0.0037 (10)
O91	0.102 (2)	0.0302 (13)	0.083 (2)	0.000	0.0377 (18)	0.000
C92	0.0398 (17)	0.0344 (16)	0.0376 (19)	0.000	0.0125 (13)	0.000
C91	0.0423 (14)	0.0741 (19)	0.0455 (16)	−0.0120 (13)	0.0003 (11)	0.0037 (13)

### Geometric parameters (Å, °)

**Table:**

S2—C8^i^	1.7591 (19)	C9—C9^i^	1.427 (3)
S2—C8	1.7591 (19)	C9—C10	1.487 (2)
S1—C1	1.716 (2)	C7—H7	0.9500
S1—C6	1.7305 (19)	C11—C12	1.504 (3)
O1—C2	1.376 (2)	C11—H11A	0.9900
O1—C3	1.441 (2)	C11—H11B	0.9900
O4—C10	1.345 (2)	C1—C2	1.352 (3)
O4—C11	1.450 (2)	C1—H1	0.9500
O2—C5	1.362 (2)	C3—H3A	0.9900
O2—C4	1.449 (2)	C3—H3B	0.9900
C8—C9	1.373 (2)	C12—H12A	0.9800
C8—N1	1.378 (2)	C12—H12B	0.9800
N1—C7	1.287 (2)	C12—H12C	0.9800
C6—C5	1.374 (2)	O91—C92	1.209 (4)
C6—C7	1.430 (2)	C92—C91	1.482 (3)
O3—C10	1.206 (2)	C92—C91^ii^	1.482 (3)
C5—C2	1.421 (2)	C91—H91A	0.9800
C4—C3	1.508 (3)	C91—H91B	0.9800
C4—H4A	0.9900	C91—H91C	0.9800
C4—H4B	0.9900		
			
C8^i^—S2—C8	91.11 (12)	C12—C11—H11A	110.5
C1—S1—C6	92.09 (9)	O4—C11—H11B	110.5
C2—O1—C3	110.73 (14)	C12—C11—H11B	110.5
C10—O4—C11	116.47 (15)	H11A—C11—H11B	108.7
C5—O2—C4	111.74 (14)	C2—C1—S1	111.79 (15)
C9—C8—N1	123.57 (16)	C2—C1—H1	124.1
C9—C8—S2	111.18 (14)	S1—C1—H1	124.1
N1—C8—S2	125.22 (14)	C1—C2—O1	124.73 (17)
C7—N1—C8	122.18 (17)	C1—C2—C5	112.87 (17)
C5—C6—C7	129.44 (18)	O1—C2—C5	122.40 (17)
C5—C6—S1	110.36 (14)	O1—C3—C4	110.85 (16)
C7—C6—S1	120.20 (14)	O1—C3—H3A	109.5
O2—C5—C6	123.48 (17)	C4—C3—H3A	109.5
O2—C5—C2	123.63 (17)	O1—C3—H3B	109.5
C6—C5—C2	112.89 (17)	C4—C3—H3B	109.5
O2—C4—C3	110.95 (16)	H3A—C3—H3B	108.1
O2—C4—H4A	109.4	C11—C12—H12A	109.5
C3—C4—H4A	109.4	C11—C12—H12B	109.5
O2—C4—H4B	109.4	H12A—C12—H12B	109.5
C3—C4—H4B	109.4	C11—C12—H12C	109.5
H4A—C4—H4B	108.0	H12A—C12—H12C	109.5
C8—C9—C9^i^	113.26 (10)	H12B—C12—H12C	109.5
C8—C9—C10	122.85 (16)	O91—C92—C91	122.52 (15)
C9^i^—C9—C10	123.63 (10)	O91—C92—C91^ii^	122.52 (15)
O3—C10—O4	123.50 (18)	C91—C92—C91^ii^	115.0 (3)
O3—C10—C9	126.89 (18)	C92—C91—H91A	109.5
O4—C10—C9	109.60 (15)	C92—C91—H91B	109.5
N1—C7—C6	119.26 (18)	H91A—C91—H91B	109.5
N1—C7—H7	120.4	C92—C91—H91C	109.5
C6—C7—H7	120.4	H91A—C91—H91C	109.5
O4—C11—C12	105.98 (17)	H91B—C91—H91C	109.5
O4—C11—H11A	110.5		
			
C8^i^—S2—C8—C9	−0.34 (9)	C8—C9—C10—O3	−45.5 (3)
C8^i^—S2—C8—N1	−178.4 (2)	C9^i^—C9—C10—O3	140.6 (2)
C9—C8—N1—C7	177.54 (18)	C8—C9—C10—O4	133.27 (18)
S2—C8—N1—C7	−4.6 (3)	C9^i^—C9—C10—O4	−40.6 (3)
C1—S1—C6—C5	0.26 (16)	C8—N1—C7—C6	−176.81 (17)
C1—S1—C6—C7	179.57 (16)	C5—C6—C7—N1	−177.75 (19)
C4—O2—C5—C6	166.96 (18)	S1—C6—C7—N1	3.1 (3)
C4—O2—C5—C2	−13.4 (3)	C10—O4—C11—C12	−173.97 (17)
C7—C6—C5—O2	0.3 (3)	C6—S1—C1—C2	−0.26 (16)
S1—C6—C5—O2	179.49 (14)	S1—C1—C2—O1	−179.73 (14)
C7—C6—C5—C2	−179.43 (19)	S1—C1—C2—C5	0.2 (2)
S1—C6—C5—C2	−0.2 (2)	C3—O1—C2—C1	161.14 (19)
C5—O2—C4—C3	43.3 (2)	C3—O1—C2—C5	−18.8 (2)
N1—C8—C9—C9^i^	179.0 (2)	O2—C5—C2—C1	−179.69 (17)
S2—C8—C9—C9^i^	0.9 (3)	C6—C5—C2—C1	0.0 (2)
N1—C8—C9—C10	4.5 (3)	O2—C5—C2—O1	0.2 (3)
S2—C8—C9—C10	−173.54 (14)	C6—C5—C2—O1	179.92 (16)
C11—O4—C10—O3	−6.8 (3)	C2—O1—C3—C4	48.8 (2)
C11—O4—C10—C9	174.33 (15)	O2—C4—C3—O1	−63.9 (2)

Symmetry codes: (i) −*x*+1, *y*, −*z*+1/2; (ii) −*x*, *y*, −*z*+3/2.

### Hydrogen-bond geometry (Å, °)

**Table:**

*D*—H···*A*	*D*—H	H···*A*	*D*···*A*	*D*—H···*A*
C1—H1···O1^iii^	0.95	2.58	3.514 (3)	168 (2)

Symmetry codes: (iii) −*x*, −*y*, −*z*−1.
